# Single-Cell Transcriptomics-Based Study of Transcriptional Regulatory Features in the Non-Obstructive Azoospermia Testis

**DOI:** 10.3389/fgene.2022.875762

**Published:** 2022-05-20

**Authors:** Xiao-juan Tang, Qiao-hong Xiao, Xue-lin Wang, Yan He, Ya-nan Tian, Bin-tong Xia, Yang Guo, Jiao-long Huang, Peng Duan, Yan Tan

**Affiliations:** ^1^ Department of Andrology, Renmin Hospital, Hubei University of Medicine, Shiyan, China; ^2^ Key Laboratory of Zebrafish Modeling and Drug Screening for Human Diseases of Xiangyang City, Department of Obstetrics and Gynaecology, Xiangyang No. 1 People’s Hospital, Hubei University of Medicine, Xiangyang, China; ^3^ Postgraduate Training Basement of Jinzhou Medicical University, Xiangyang No.1 People’s Hospital, Hubei University of Medicine, Xiangyang, China; ^4^ Department of Urology Surgery, Xiangyang No.1 People’s Hospital, Hubei University of Medicine, Xiangyang, China; ^5^ Hubei Key Laboratory of Embryonic Stem Cell Research, Hubei University of Medicine, Shiyan, China; ^6^ Biomedical Engineering College, Hubei University of Medicine, Shiyan, China

**Keywords:** non-obstructive azoospermia, spermatogenesis, leydig cells, testicular macrophages, transcription factors

## Abstract

Non-obstructive azoospermia (NOA) is one of the most important causes of male infertility. Although many congenital factors have been identified, the aetiology in the majority of idiopathic NOA (iNOA) cases remains unknown. Herein, using single-cell RNA-Seq data sets (GSE149512) from the Gene Expression Omnibus (GEO) database, we constructed transcriptional regulatory networks (TRNs) to explain the mutual regulatory relationship and the causal relationship between transcription factors (TFs). We defined 10 testicular cell types by their marker genes and found that the proportion of Leydig cells (LCs) and macrophages (tMΦ) was significantly increased in iNOA testis. We identified specific TFs including LHX9, KLF8, KLF4, ARID5B and RXRG in iNOA LCs. In addition, we found specific TFs in iNOA tMΦ such as POU2F2, SPIB IRF5, CEBPA, ELK4 and KLF6. All these identified TFs are strongly engaged in cellular fate, function and homeostasis of the microenvironment. Changes in the activity of the above-mentioned TFs might affect the function of LCs and tMΦ and ultimately cause spermatogenesis failure. This study illustrate that these TFs play important regulatory roles in the occurrence and development of NOA.

## Introduction

Male infertility accounts for almost half of all infertility cases and is considered a common, multifactorial pathological condition resulting from a combination of genetic, environmental and lifestyle factors (Kuroda et al., 2020; Sharma et al., 2021). The genetic landscape of male infertility has not been well defined likely due to technical challenges. A substantial proportion of male infertility is accompanied by azoospermia, usually manifested as non-obstructive azoospermia (NOA), which affects about 1% of men in the general population (Tournaye et al., 2017; Zhao et al., 2020; Salas-Huetos et al., 2021). NOA is defined as the complete absence of spermatozoa during ejaculation as a result of failed spermatogenesis. Currently, NOA remains the most challenging and clinically severe form of male infertility and it is primarily associated with genetic abnormalities (Kuroda et al., 2020; Han et al., 2021). A relatively small proportion of NOA is caused by congenital factors such as Klinefelter syndrome (KS) and microdeletions in the azoospermia factor (AZF) region of the Y chromosome (Kuroda et al., 2020; Sharma et al., 2021). However, a majority of the remaining NOA cases have idiopathic (unknown) causes and are diagnosed as idiopathic NOA (iNOA), which accounts for more than 70% of cases (Dabaja and Schlegel, 2013; Zhao et al., 2020; Salas-Huetos et al., 2021). Although there have been some advances in understanding the aetiology and pathogenesis of NOA (including inherent testicular injury or gonadotropin deficiency), the transcriptional regulatory network (TTRN) in spermatogenesis as well as NOA remains not fully clear.

Spermatogenesis is regulated by cross-talk between somatic cells and germ cells in the testis (Meinhardt et al., 2018; Winge et al., 2018; Zhou et al., 2019; Zhao et al., 2020; Hashimoto et al., 2021). Testicular somatic cells, including Leydig cells (LCs) and macrophages, interact with each other to create a supportive microenvironment for germ cell development and self-renewal of spermatogonial stem cells (SSCs), both of which are indispensable for spermatogenesis and male fertility (DeFalco et al., 2015; Teerds and Huhtaniemi, 2015; Mossadegh-Keller et al., 2017; Jauregui et al., 2018; Meinhardt et al., 2018; Winge et al., 2018; Zhou et al., 2019; Lokka et al., 2020; Figueiredo et al., 2021; Hashimoto et al., 2021). In particular, LCs and testicular macrophages (tMΦ) are both located in the testicular interstitial compartment and are functionally related (DeFalco et al., 2015; Mossadegh-Keller et al., 2017; Lokka et al., 2020; Hashimoto et al., 2021). LCs are the primary cells responsible for synthesising and releasing androgens, and these hormones regulate both spermatogenesis and the development of male-specific secondary sex characteristics. tMΦ not only sustain an immune-privileged microenvironment but also engage in collaborative interactions with LCs (Meinhardt et al., 2018; Mossadegh-Keller and Sieweke, 2018; Figueiredo et al., 2021). Increasing evidence indicates that alteration in somatic cell function or the somatic microenvironment could hinder spermatogenesis and lead to NOA (Zhao et al., 2020; Zheng et al., 2021b; Hauptman et al., 2021; Zhu et al., 2021). Nevertheless, the mechanism by which somatic cells contribute to spermatogenesis, particularly the somatic cells in the testicular iNOA microenvironment, is still poorly understood.

More recently, it has emerged that, even after cell development and differentiation, the maintenance of adult somatic cell identity and function relies on the continuous activity of transcription factors (TFs) (Shan et al., 2017). Some TFs could affect not only cell function through expression, but could also regulate cell differentiation (Ieda et al., 2010; Riddell et al., 2014; Han et al., 2018). An increasing number of TFs in testicular somatic cells have been found to regulate a variety of fundamental cell functions during spermatogenesis and testis development (Meroni et al., 2019; Zhou et al., 2019; Jia et al., 2020; Uchida et al., 2020; Wang et al., 2020; Sarkar et al., 2021). However, it remains largely unclear how TFs in LCs and tMΦ play an active role in the development of NOA. In this study, we used the complete atlas of human testicular single cell data to construct transcriptional regulatory networks (TRNs) in the iNOA testis (Zhao et al., 2020). For this endeavour, we used single-cell transcriptome data in conjunction with the gene regulatory network approach. We initially defined TFs influencing the testicular somatic TRNs, revealing that LCs and tMΦ have specific TRNs in the NOA testis.

## Materials and Methods

As our study was based on a conjoint analysis of existing data and no additional patients were included. Hence, ethical approval was not required.

### Datasets

We downloaded a human testicular single cell RNA-Seq data set (GSE149512) from the Gene Expression Omnibus (GEO). We selected three normal adults (GSM4504189, GSM4504187 and GSM4504184) and three patients with iNOA (GSM4504195, GSM4504196 and GSM4504197). The quality control (QC) of single-cell RNA-Seq data was performed by using the scater package in R (McCarthy et al., 2017). Genes expressed in at least 2 cells were retained. Mitochondrial (MT) genes were set as the internal reference. For cells with total counts <25,000 or total genes >6,000, the percentage of MT genes >40 were removed. The scImpute package in R was used for imputation, and normalisation was conducted by using the scran package in R (Vieth et al., 2019). RNA-Seq data were normalised by using the transcripts per kilobase million (TPM) method for further analysis.

### Dimensional Reduction and Clustering

We performed principal component analysis (PCA) together with JackStraw and PCEIbow-Plot functions by using the Seurat package (version 3.2.2) in R (version 4.0.2), to select important principal components (PCs) (Lin et al., 2017; Butler et al., 2018). Seurat’s Find All-Markers function was used to identify specific genes for each cell subpopulation. The Run TSNE function was then used for cell clustering and visual analysis of t-distributed stochastic neighbour embedding (t-SNE). The marker genes were thereafter annotated with the singleR package and corrected with CellMarker according to their characteristics (Aran et al., 2019; Zhang et al., 2019). Then, heatmaps were made up of the first 50 marker genes in each cell population, and Gene Ontology (GO) terms were selected to represent the function of each cell type with *p* < 0.05 among top 30 terms.

### Gene Set Variation Analysis

We performed GSVA to reveal the underlying changes in signalling mechanisms using R (Hanzelmann et al., 2013). The differentially expressed Kyoto Encyclopedia of Genes and Genomes (KEGG) pathways were identified from the data of the normal and iNOA groups. The gene set c2. cp.kegg.v7.2. symbols.gmt was downloaded from the Molecular Signatures Database (MSigDB) and set as the reference gene list (Liberzon et al., 2015). After inputting the gene expression profile matrix, the GSVA algorithm transformed the genes of the matrix into scores that represented the activity of each KEGG pathway based on the reference gene. Then, the differentially activated pathways between the normal and iNOA groups were determined by using the limma package in R with |log2fold change| ≥ 2 and *p* < 0.05 (Ritchie et al., 2015).

### Inference of Regulons and Activity

A number of methods have been developed to predict genetic regulatory networks (GRNs) from single-cell gene expression data. We adopted the SCENIC method as previously described with slight modification (Fiers et al., 2018). The SCENIC analytic process, comprised three steps. First, a gene co-expression network was constructed through gene co-expression analysis. Second, possible TF-target regulatory relationships were established based on the gene co-expression network. In this step, the direct regulatory relationship was established by using motif analysis. Any direct downstream genes occurring for each TF were profiled as regulons. In particular, SCENIC could only support transcriptional positive regulation analysis. Third, based on the results of step 2, a regulon activity score (RAS) was calculated for each cell. As described in previous studies, the Avg20 method was repeated three times to assess the variability of random sampling. Thereafter, a *t*-test was used to assess whether the Avg20 method performed better than using all individual cells (Cao et al., 2017).

### Functional Validation

As in a previous study, we used Search-Based Exploration of Expression (SEEK) analysis to determine whether the predicted regulons correlated with their cell type (Zhu et al., 2015). In brief, we used the human version of SEEK to assess whether genes in the regulons were co-expressed. Significantly co-expressed genes in multiple data sets associated with a particular cell type scored positive for high relevance of the function of the regulon to that cell type.

### Regulon Module Analysis and Quantifying Cell Type Relationship

To identify regulon modules, we employed two main steps (Aibar et al., 2017). First, each pair of regulatory relationships was analysed by calculating the Pearson correlation coefficient. To systematically describe the regulatory relationships of TFs, we compared the regulatory activity scores of each regulatory pair based on the connection-specificity index (J. I. F. Bass et al., 2013). For each pair of regulatory relationships, we defined a regulatory specificity score (RSS) based on the Jensen–Shannon scatterplot (Cabili et al., 2011). Next, we selected the specific regulator with the highest RSS value and further examined its functional characteristics. The activity score of each regulon module in relation to a cell type was then defined as the average of the activity scores of its regulon members in all cells of that cell type. The highest ranked units were then filtered for each module. We quantified the relationship between different cell types based on the similarity of overall regulon activity. A pair of cell types was linked if their Spearman correlation coefficient was >0.8. Finally, we used the Markov Clustering Algorithm (MCL) to identify related cell types (Van Dongen and Abreu-Goodger, 2012).

### GO, KEGG Enrichment Analysis and Protein-Protein Interaction Networks

We conducted GO enrichment analysis and KEGG analysis on the TF of each regulatory module example. GO analysis depicts the unique biological significance based on differentially expressed genes (DEGs) between groups. We used the KEGG database to determine important pathways. The ‘*p* < 0.05’ and the ‘|log2 fold change| ≥ 2’ conditions were used as the cut-off criteria for GO and KEGG enrichment analyses. The genes of each module are then incorporated into a search tool (STRING) (Jensen et al., 2009) that retrieves interacting genes/proteins. In the multivariate analysis, the confidence levels were 0.4, 0.7 and 0.9. Then, we input the gene network file into Cytoscape to draw PPIN diagrams.

## Results

### Single-Cell Maps Define the Heterogeneity of Normal and iNOA Testicular Cells

**FIGURE 1:**
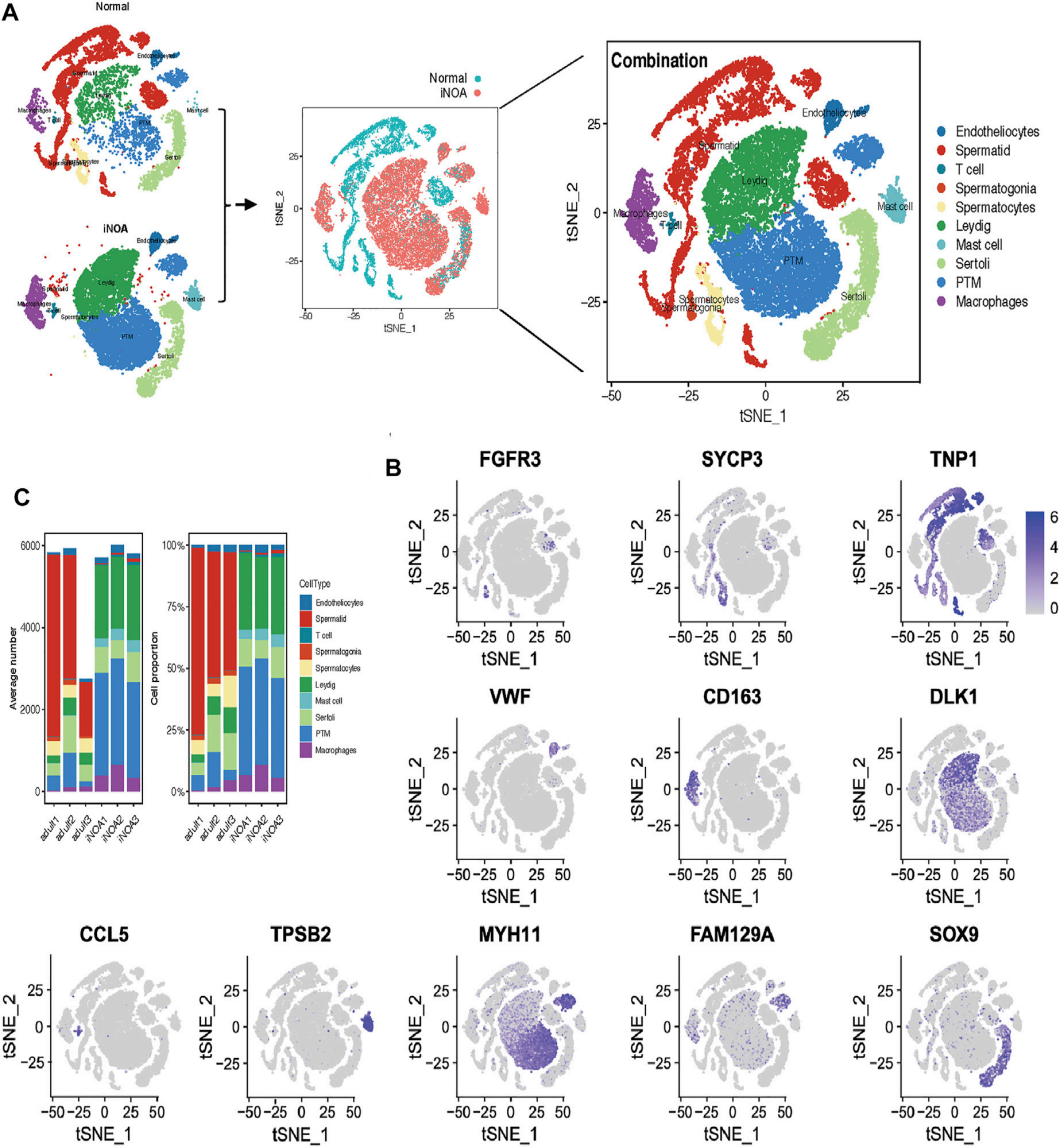
Single-cell RNA sequencing (scRNA-Seq) analysis shows the testicular cell lineages in normal individuals and patients with idiopathic non-obstructive azoospermia (iNOA). **(A)** t-Distributed random neighbour embedding (t-SNE) isolation (left) and combination (middle and right) of single testicular cells in the normal and iNOA groups. Ten main cell types were defined, including endotheliocytes, T cells, mast cells, Leydig cells, Sertoli cells, peritubular myoid (PTM) cells, macrophages, spermatogonia, spermatocytes and spermatids. **(B)** The t-SNE map shows the expression level distribution of marker genes in cell types, including FGFR3 (spermatogonia), SYCP3 (spermatocytes), TNP1 (spermatids), VWF (endotheliocytes), CD163 (macrophages), DLK (Leydig cells), MYH11 (PTM cells), SOX9 (Sertoli cells), CCL5 (T cells) and TPSB2 (mast cells). **(C)** The mean cell number and relative proportion of testicular subsets from various sample sources.

To investigate the heterogeneity of patients with iNOA, we used the Seurat package to perform quality control and t-SNE analysis on single-cell data from the GEO (GSE149512) data set. 32,048 cells, each with 500–8,000 genes, were reserved for subsequent analysis. We divided the normal and iNOA groups into 10 cell populations based on marker genes of each cell population (Figures 1A,B, Supplementary Figure S1). The cellular composition of the iNOA group was different compared qwith the normal group. Almost no sperm cells were observed in the iNOA group, while the proportion of LCs and tMΦ were increased significantly in the iNOA group compared with the normal group (Figure 1C).

### Marker Gene GO Analysis

**FIGURE 2:**
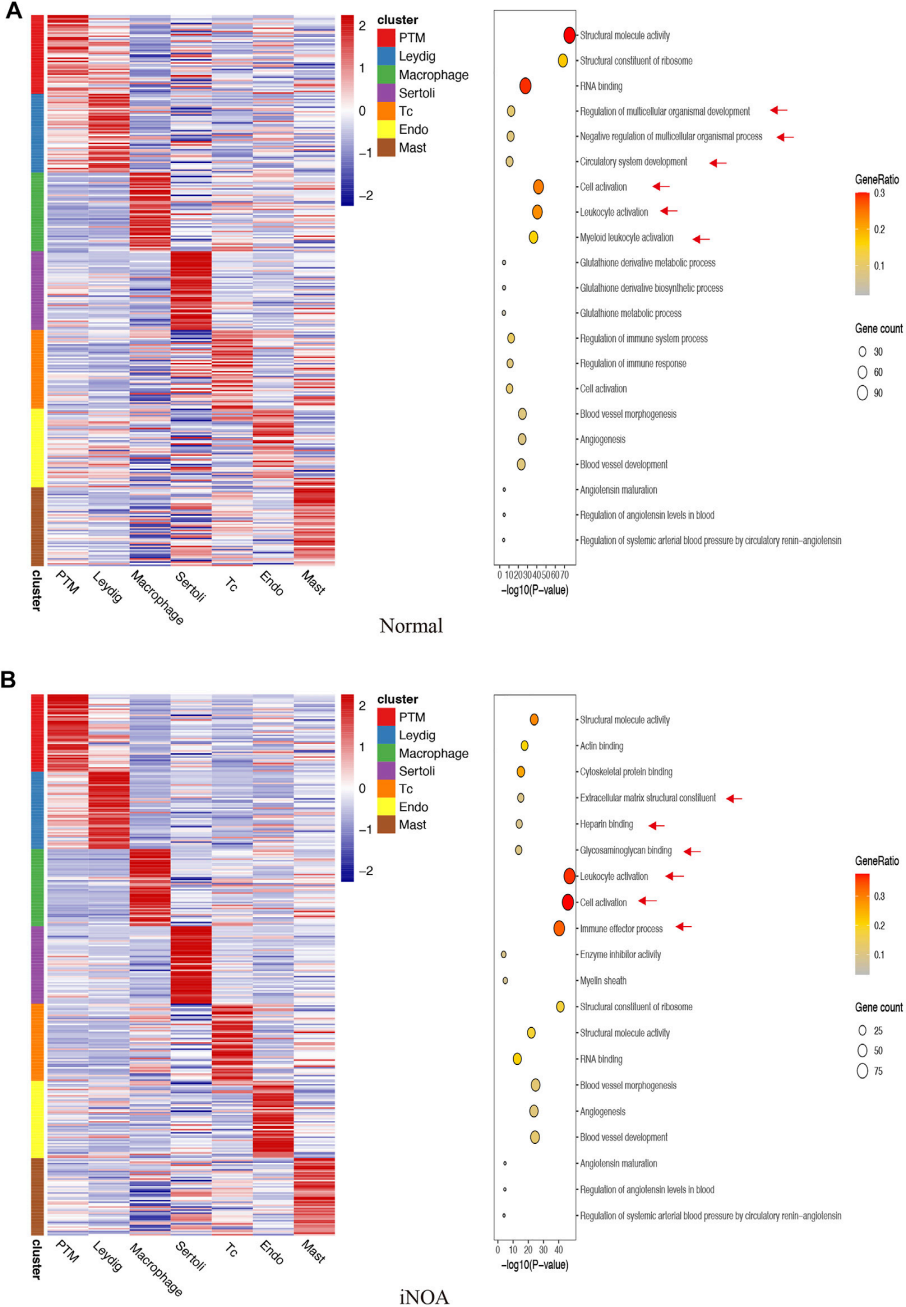
Gene Ontology (GO) enrichment analysis of marker genes in **(A)** the normal group and **(B)** the idiopathic non-obstructive azoospermia (iNOA) group. Left: heatmap showing expression signatures of the top 50 specifically expressed genes in each cell type; the value for each gene is the row-scaled Z score. Right: representative GO terms.

We identified unique characteristics of the iNOA group. For example, normal LC GO terms include ‘Regulation of multicellular organismal development’ and ‘Negative Regulation of multicellular cells’ organismal process (Figure 2A), while iNOA group LCs GO terms include ‘Extracellular matrix structural constituent’ and ‘Glycosaminoglycan binding’ (Figure 2B). The normal tMΦ GO terms include ‘Myeloid leukocyte activation’, while iNOA tMΦ GO terms include ‘Immune effector process’ (Figures 2A,B). These results indicate that the functions of LCs and tMΦ are different between the normal and iNOA groups.

### GSVA Analysis of Testicular Cells

**FIGURE 3:**
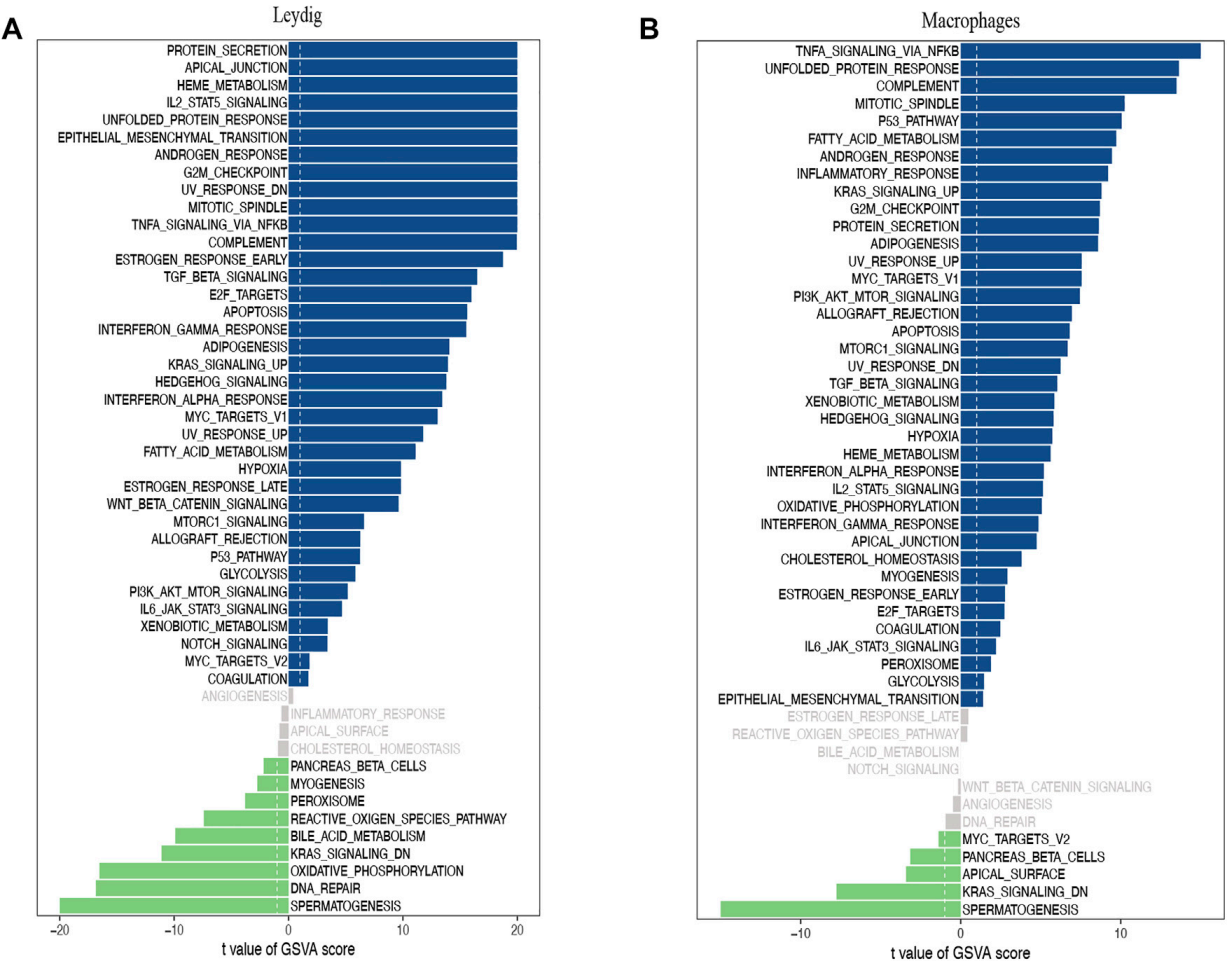
Gene set variation analysis (GSVA) of testicular somatic cells in the normal group and the idiopathic non-obstructive azoospermia (iNOA) group. **(A)** Differences in pathway activities scored per cell by GSVA between normal and iNOA Leydig cells (n = 968 and 9,412 cells, respectively; six patients per group). **(B)** Differences in pathway activities scored per cell by GSVA between normal and iNOA macrophages (n = 144 and 763 cells, respectively; six patients per group).

A direct comparison of the iNOA and normal groups revealed that ‘G2M checkpoint’, ‘MTORC1 signaling’ and ‘TGF beta signaling’ as the top enriched signatures in iNOA LCs, are associated with proliferation (Figure 3A). Moreover, the GSVA scores of the ‘mitotic spindle’, ‘MTORC1 signaling’ and ‘TGF beta signaling’ were obviously increased in iNOA tMΦ, and these pathways are also associated with proliferation (Figure 3B). We found that the spermatogenesis pathways were obviously downregulated in the iNOA group including LCs, tMΦ, peritubular myoid (PTM) cells and Sertoli cells compared with the normal group (Figures 3A,B, Supplementary Figure S2).

### Analysis of Specific Regulation of Somatic Cell Types

**FIGURE 4:**
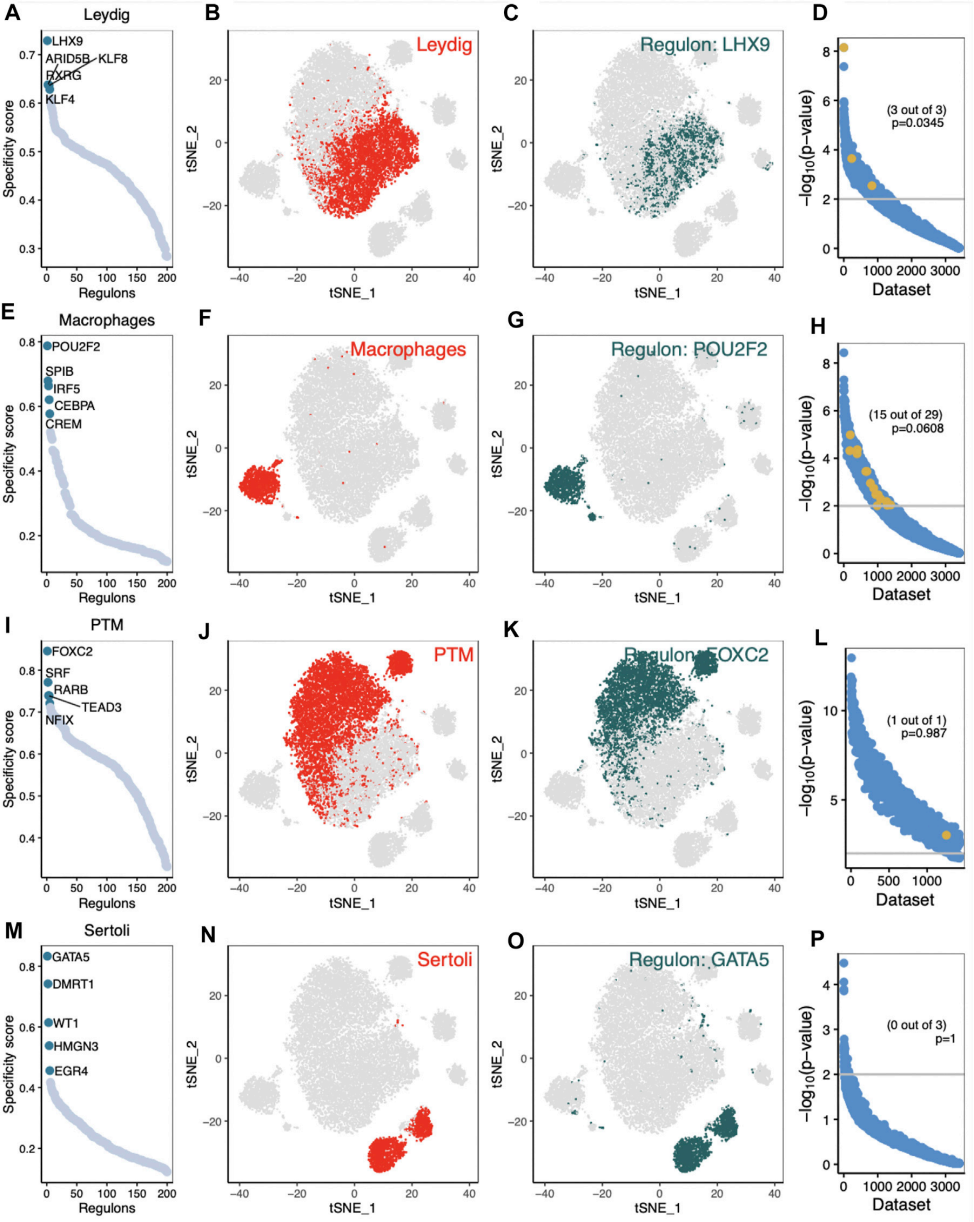
Analysis of cell type-specific regulation in testis of idiopathic non-obstructive azoospermia (iNOA) patients. **(A–D)** Leydig cells (LCs). **(A)** The regulation of testicular cells was ranked according to the regulation specificity score. **(B)** LCs are marked by red dots on the t-distributed random neighbour embedding (t-SNE) diagram. **(C)** The expression values of the genes with the highest regulatory activity score are displayed in the t-SNE diagram. **(D)** Search-Based Exploration of Expression (SEEK) analysis was used to determine the co-expression of the highest regulatory genes in different Gene Expression Omnibus (GEO) data sets. The *x*-axis represents the different data sets, and the *y*-axis represents the co-expression significance of the target gene in each data set. Data sets with a significant correlation (*p* < 0.05) are highlighted with yellow dots. **(E–H)** The same as **(A–D)**, but for testicular macrophages. **(I–L)** The same as **(A–D)**, but for peritubular myoid (PTM) cells. **(M–P)** The same as **(A–D)**, but for Sertoli cells.

Our network analysis found that NR2F1, CREB3l1, HC1, GLI2 and KLF4 were specific TFs associated with LCs in normal human (Supplementary Figure S3A). The t-SNE diagrams further demonstrated the highly specific activity of NR2F1 in LCs (Supplementary Figure S3B, C). To test the validity of the above analysis, SEEK analysis was used to identify GEO data sets with significant co-expression of NR2F1. SEEK analysis did not show significant co-expression of the regulatory gene NR2F1 (Supplementary Figure S3D, Fisher’s exact test, *p* = 0.0858). CEBPA, MEF2A, IRF5, HIVEP2 and EOMES were found to be specific TFs in normal tM φ (Supplementary Figure S3E). CEBPA activity was highly specific in normal tMΦ (Supplementary Figure S3F, G). SEEK analysis did not show significant co-expression of the regulatory gene CEBPA (Supplementary Figure S3H, Fisher’s exact test, *p* = 1). Other specific regulation of cell types is shown in Supplementary Figure S3I–P; Furthermore, our network analysis identified LHX9, ARID5B, KLF8, RXRG and KLF4 as specific TFs associated with iNOA LCs, and found that the activity of these TFs was elevated in iNOA patients (Figure 4A). The t-SNE diagram further demonstrated the highly specific activity of LHX9 in iNOA LCs (Figures 4B,C). To test the validity of the above analysis, we performed SEEK analysis to identify GEO data sets with significant co-expression of LHX9 (Figure 4D, Fisher’s exact test, *p* = 0.0345). We identified POU2F2, SPIB, IRF5, CEBPA and CREM as specific TFs in iNOA tMΦ, and the activity of these TFs was also elevated in iNOA patients (Figure 4E). The activity of POU2F2 in iNOA tMΦ was highly specific (Figures 4F,G). SEEK analysis did not show significant co-expression of the regulatory gene POU2F2 (Figure 4H, Fisher’s exact test, *p* = 0.0608). For other specific regulation of cell types, see Figure 4I–P; for specific regulation of cell types in the normal group, see Supplementary Figure S3.

### Organizing Regulons Into Combinatorial Modules

**FIGURE 5:**
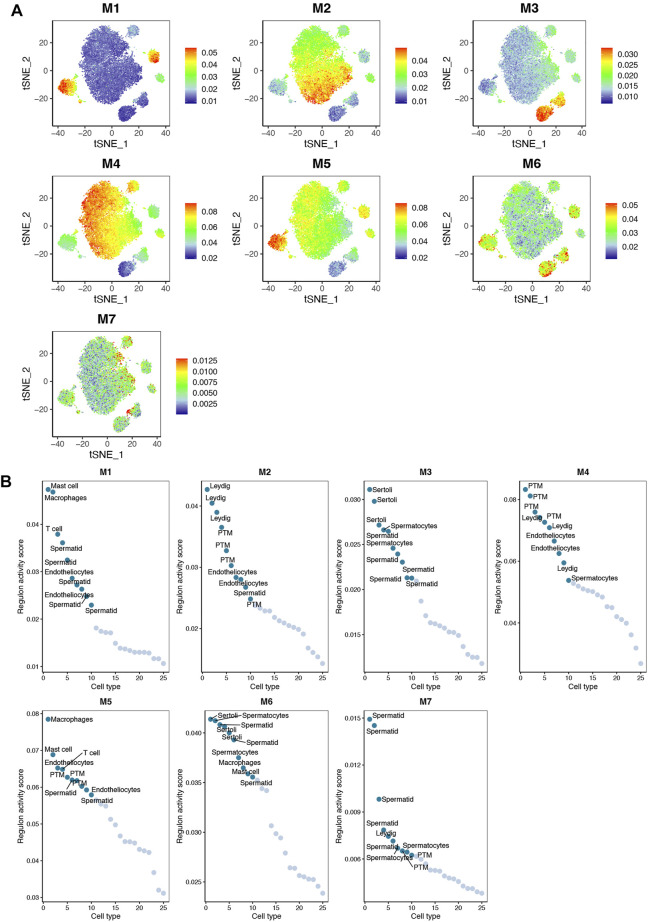
The activity of regulatory modules in testicular somatic cells of different idiopathic non-obstructive azoospermia (iNOA) types. **(A)** Identification of the regulatory modules (M1–M7) according to the regulatory connection specificity index (CSI) matrix and a map of the average activity of each module based on t-distributed random neighbour embedding (t-SNE). **(B)** Ranking regulation in iNOA testicular cells based on the regulation specificity score. The *y*-axis represents the regulation activity score. The *x*-axis represents the cell type.

**FIGURE 6:**
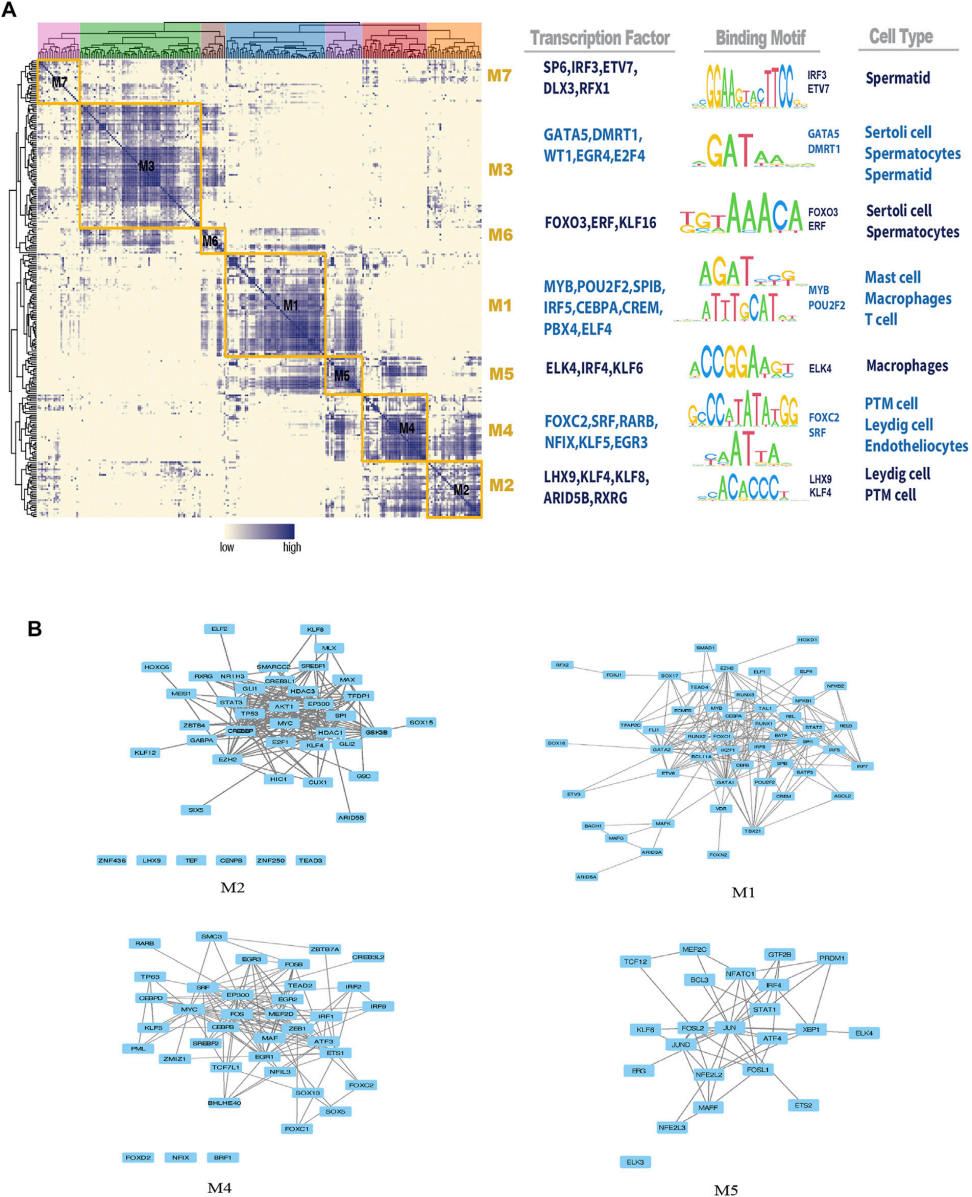
Identification of combinatorial regulon modules. **(A)** Determination of the regulon modules based on the regulation connection specificity index (CSI) matrix, along with associated cell types, corresponding binding motifs, and representative transcription factors (TFs). **(B)** Protein-protein interaction networks (PPINs) of regulatory factors in each module.

Based on the identified connection specificity index (CSI) matrix model (M1–M7), we mapped the average activity of each module to t-SNE (Figure 5A). The iNOA testicular cells were then ranked according to the regulatory specificity score (Figure 5B). Each module occupies a different region, and all the highlighted regions indicate the high transcriptional activity of different modules (Figure 5A). As shown in Figure 6A, the M1 and M5 modules showed high transcriptional activity mainly in iNOA tMΦ, while the M2 and M4 modules showed high transcriptional activity mainly in iNOA LCs. Figure 6A showed the determination of the regulation module based on the regulation CSI matrix, along with associated cell types, corresponding binding motifs, and representative transcription factors. The PPINs of TFs in each module were shown in Figure 6B. The M1 module contains MYB, POU2F2 and PBX4 in iNOA tMΦ. In addition, in iNOA LCs the M2 and M4 modules contain LHX9, KLF8, KLF4 and FOXC2. We performed GO functional enrichment analysis on genes of the M1, M2, M4 and M5 modules, as shown in Supplementary Figure S4.

## Discussion

Spermatogenesis is a highly sophisticated process with well-organised cellular and molecular events involving gene networks regulated by TFs in testicular somatic cells (Bettegowda and Wilkinson, 2010). Our results also support the view that the somatic cellular microenvironment provides favourable biochemical and biophysical components for spermatogenesis. We focussed on LCs and tMΦ in the testicular microenvironment, which are critical for sperm production, and identified some important TFs in LCs and tMΦ that could play an important role in NOA genesis.

Unlike obstructive azoospermia, the pathogenesis of iNOA remains complex and multifactorial. To study the molecular mechanism of iNOA pathogenesis, we analysed single-cell data sets from the GEO database, and clarified the heterogeneity of different cell types of the human testis in more detail by using single-cell transcriptome sequencing analysis. We found that the proportion of LCs and tMΦ in iNOA testis is higher than the normal group. Furthermore, the function of LCs and tMΦ in the iNOA testis is different from the normal group. We suggest that changes in the proportions and functions of LCs and tMΦ could alter the testicular microenvironment, contributing to spermatogenesis failure and thereby leading to male infertility.

Testicular LCs are the major producers of circulating testosterone, which is essential for testis development and spermatogenesis. LC dysfunction can lead to testosterone deficiency and impair male fertility (Zhou et al., 2019). The function of LCs is correlated with the developmental stages of LC lineage specification and differentiation, both as Sertoli cells and tMΦ. LC development involves at least three steps: the proliferation of LC precursors (also known as stem LCs), their differentiation into immature LCs and their final maturation into adult LCs (Peak et al., 2016). We suppose that LCs of patients with iNOA remain at the stage of proliferation without transitioning to the differentiation and maturation stages. These immature and non-functional LCs are unable to (fully) fulfil their steroidogenic function to maintain spermatogenesis (O'Hara et al., 2015; Guan et al., 2019). The successive stages of LC development are regulated by an array of lineage-specific TFs (Winge et al., 2018). We identified specific TFs including LHX9, KLF8, KLF4, ARID5B and RXRG in LCs of patients with iNOA. Among these TFs, LHX9 is an important steroidogenesis-related TF and indispensable for testis development (Hu et al., 2018). KLF4 and KLF8 are Krüppel-like factors known to regulate several biological processes, such as cell proliferation, differentiation and metabolism (Chu et al., 2016; Kult et al., 2021; Kumar et al., 2021). Similarly to KLF4, ARID5B also plays a pivotal role in adipogenesis and lipid metabolism, which might be closely related to cell differentiation and development (Hata et al., 2013; Claussnitzer et al., 2015). RXRG is a rexinoid receptor that participates in the regulation of cell differentiation (Gely-Pernot et al., 2015; Cheng et al., 2018). All these identified TFs play direct/indirect roles in cell development. However, the occurrence of NOA caused by TFs has not been reported. This is our new finding. We conducted GO analysis on LCs of normal persons and iNOA patients, and found that LCs of iNOA patients were inhibited in the ‘Regulation of Multicellular Development’ pathway. GSVA enrichment analysis showed that LCs of iNOA patients were significantly enriched in the proliferation pathway, indicating that the development of LCs in iNOA patients was inhibited in the proliferation stage, but did not enter the stages of differentiation and maturity. We infer that changes in the activity of these TFs potentially affect the maturation and function of LCs and impair the microenvironment of spermatogenesis, dysfunctions that may eventually cause azoospermia in humans.

TMΦ are the principal immune cell population of the mammalian testis, and together with LCs and Sertoli cells, they maintain testicular immune privilege (Heinrich and DeFalco, 2020; Rehman et al., 2021). tMΦ produce several growth and differentiation factors for LC development (Heinrich and DeFalco, 2020). In addition to testicular immunosuppression, tMΦ also locally regulate LC steroidogenesis (Fang et al., 2021). Previous studies have delineated the phenotype of tMΦ in the normal testis, and tMΦ have been implicated in the development of azoospermia (Hussein et al., 2005; Duan et al., 2011). We identified specific TFs including POU2F2, SPIB, IRF5, CEBPA, ELK4 and KLF6 in iNOA tMΦ. Among these identified TFs, both POU2F2 and SPIB are essential for cell proliferation, differentiation and functional maturation of immune cells (Hodson et al., 2016; Klisuric et al., 2019). IRF5 has been shown to act as a master switch that promotes proinflammatory cytokine production from macrophages and thus contributes to the plasticity of macrophage polarisation (Banga et al., 2020). CEBPA is required for the regulation of cell proliferation and terminal differentiation and participates in the control of immune and inflammatory processes (Bristol et al., 2009; Minner et al., 2019). ELK4 has been implicated in maintaining cellular homeostasis, but also in macrophage M2 polarisation (Zheng et al., 2021a). Similarly to KLF4 and KLF8, KLF6 signalling engages in various cellular processes, including cell differentiation and development. Moreover, KLF6 impedes macrophage polarisation to the M2 phenotype (Zhao et al., 2021). These TFs in macrophages are newly discovered and may affect NOA. GSVA enrichment analysis of tMΦ in normal persons and iNOA patients showed that tMΦ of iNOA patients was significantly enriched in the proliferation pathway, which also indicated that thetMΦ of iNOA patients was overproliferated, which was consistent with the previous research results of Wenzhong Zheng (Zheng et al., 2021). The changes of TFs activity found in iNOA tMΦ may regulate the development and function of tMΦ and the differentiation of tM into M2 macrophages. When macrophages are activated and elaborate inflammatory mediators, the function of LCs secretion of testosterone may be impaired. (Hales, 2002), and thus fail to create an optimal immune microenvironment for spermatogenesis.

In this study, the transcriptional regulatory network of iNOA testicular cells was established to provide a new idea for understanding the regulatory mechanism and functional relationship of iNOA testicular cells. However, the roles and mechanisms of these TFs in iNOA pathogenesis in different testicular cell types need to be further investigated experimentally.

## Conclusion

With the recent application of single-cell sequencing technology in the human testis, the understanding of testicular cell heterogeneity has greatly improved. It is clear that we need more in-depth investigation of the mechanism of cellular heterogeneity during iNOA development. This study has provided a new approach to dissect the regulatory mechanisms and functional relationships by establishing the TRNs and PPINs of TFs in iNOA LCs and tMΦ. Several of the identified TFs, such as Krüppel-like factors, are predicted to regulate the differentiation and function of both LCs and tMΦ. We demonstrated that aberrant regulation of TFs identified in iNOA LCs and tMΦ potentially affects the testicular microenvironment and germ cell development. This study should improve knowledge regarding TFs involved in the regulatory landscape of LC and tMΦ development and the crosstalk among cell types. However, additional experiments are needed to investigate the function and mechanism of TFs in different testicular cell types that are involved the pathogenesis of NOA.

## Data Availability

The original contributions presented in the study are included in the article/Supplementary Material, further inquiries can be directed to the corresponding authors.
