# Supplementary material for: Single-Cell Transcriptomics-Based Study of Transcriptional Regulatory Features in the Non-Obstructive Azoospermia Testis
Source: Front Genet. 2022 May 20;13:875762. doi: 10.3389/fgene.2022.875762 (PMC9163961; doi:10.3389/fgene.2022.875762)
Supplement: Supplementary file 1 [file DataSheet1.PDF]

*Supplementary Material*

## Supplementary Figures

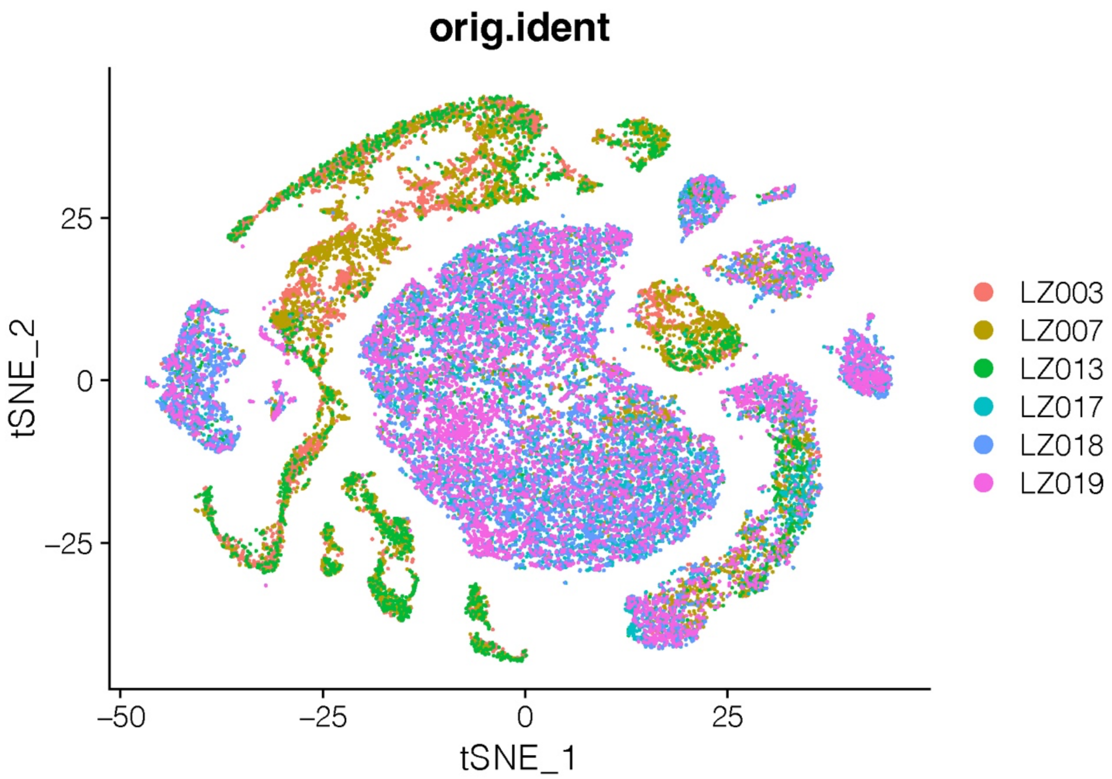


Supplementary Figure 1. t-Distributed random neighbour embedding (t-SNE) isolation

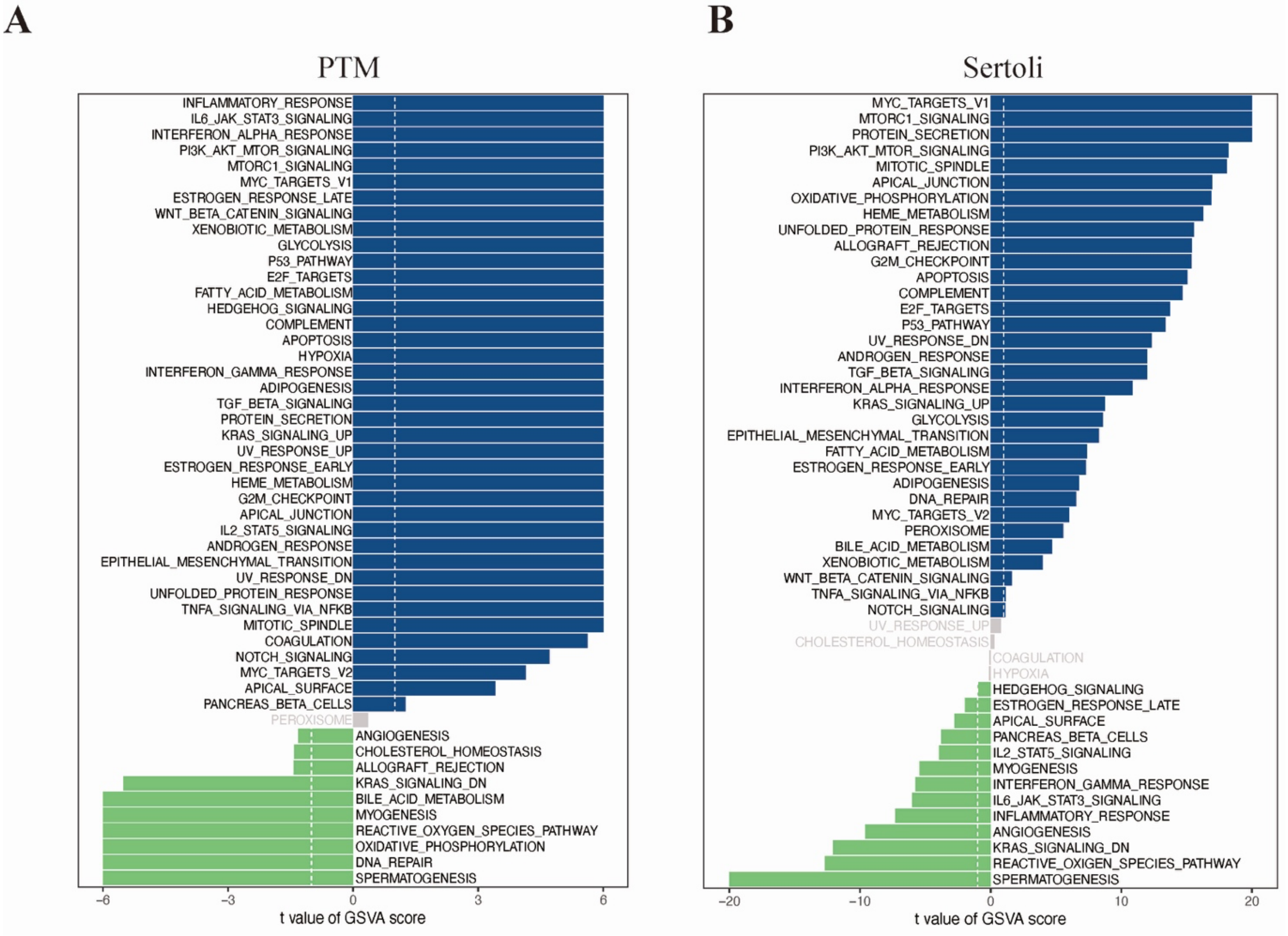


Supplementary Figure 2. Gene set variation analysis (GSVA) analysis of testicular somatic cells in the normal group and the idiopathic non-obstructive azoospermia (iNOA) group. (A) Differences in pathway activities scored per cell by GSVA between normal and iNOA peritubular myoid (PTM) cells. (B) Differences in pathway activities scored per cell by GSVA between normal and iNOA Sertoli cells.

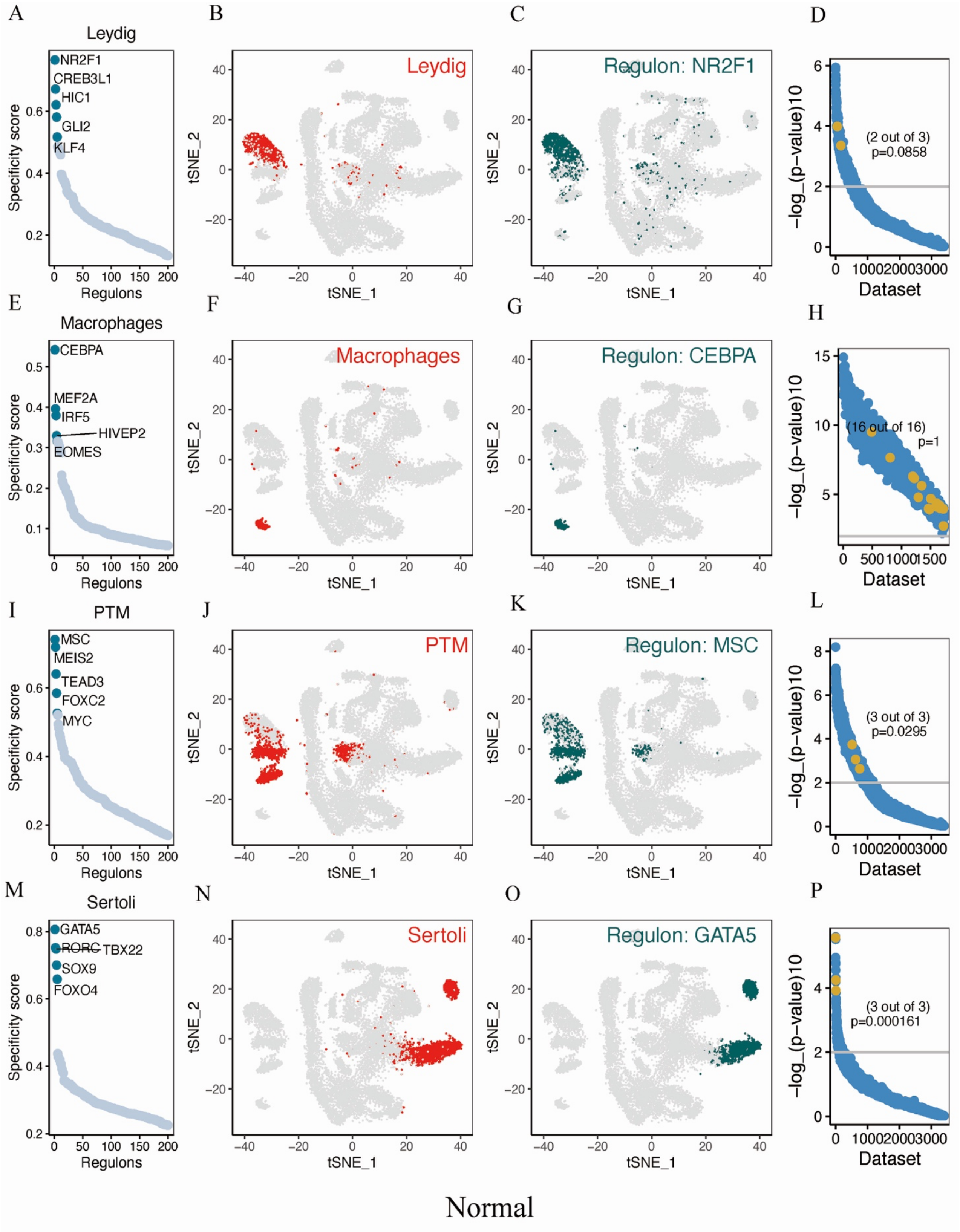


Supplementary Figure 3. Analysis of cell type-specific regulation in the testis of a normal adult: (A) Leydig cells, (B) macrophages, (C) peritubular myoid (PTM) cells and (D) Sertoli cells.

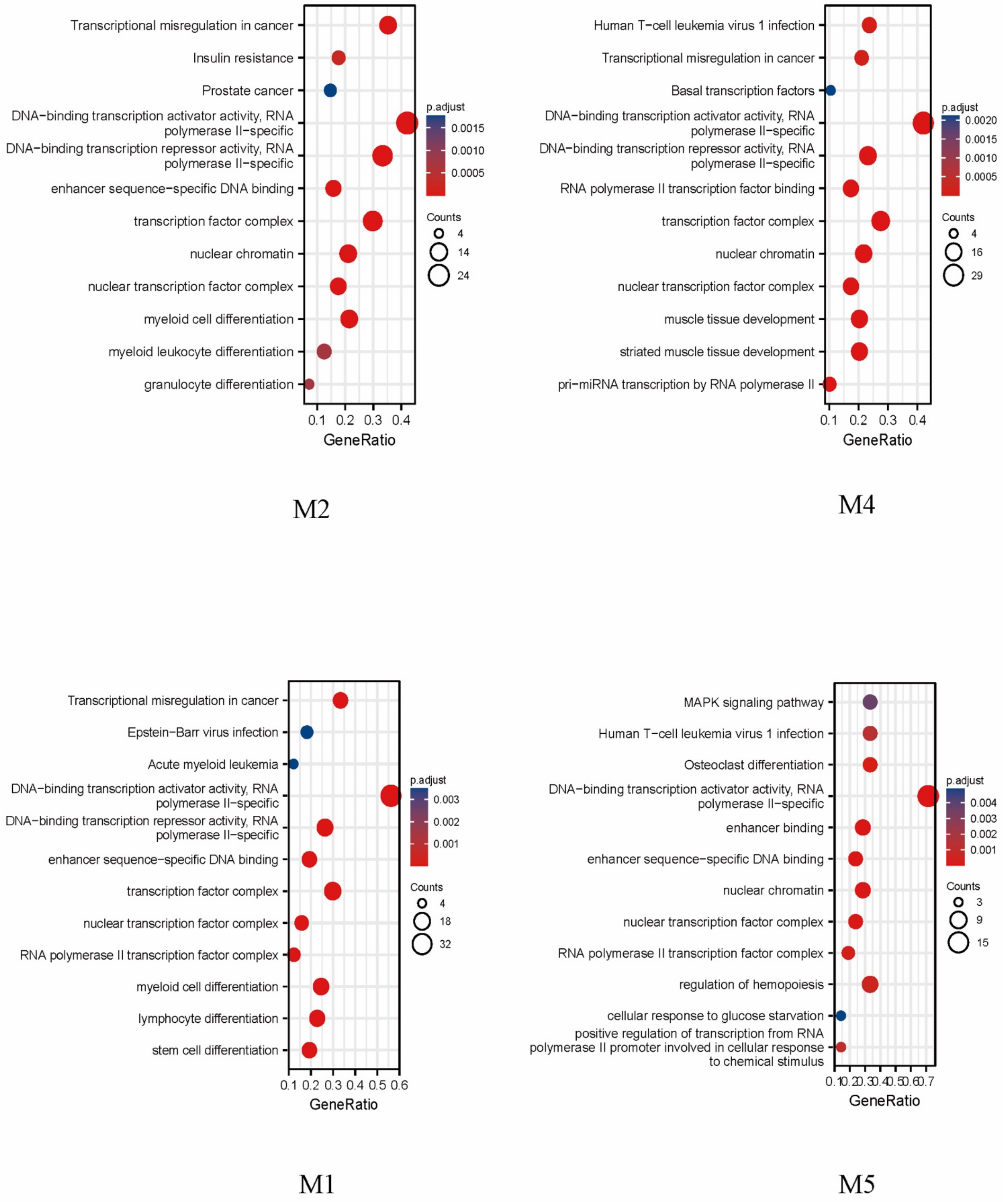


Supplementary Figure 4. Gene Ontology (GO) enrichment analysis of M1, M2, M4, and M5 module genes.
